# The concurrent validity of a portable ultrasound probe for muscle thickness measurements

**DOI:** 10.1111/cpf.12901

**Published:** 2024-09-05

**Authors:** Kai A. Homer, Matt R. Cross, Ivan Jukic

**Affiliations:** ^1^ Sport Performance Research Institute New Zealand (SPRINZ) Auckland University of Technology Auckland New Zealand; ^2^ Department of Health, Sport and Wellbeing, Faculty of Social and Applied Sciences Abertay University Dundee UK

**Keywords:** agreement, bias, imaging, Lumify, Vivid S5

## Abstract

Ultrasound imaging is extensively used by both practitioners and researchers in assessing muscle thickness (MT); however, its use in the field is constrained by the transportability of stationary devices. New portable ultrasound probes pose as a cost‐effective and transportable alternative for field‐based assessments. This study evaluated the concurrent validity of a portable probe (Lumify) against a laboratory‐based device (Vivid S5) in measuring MT. Eighteen participants (nine males and nine females) visited the laboratory and their MT measurements were collected using each device at five different sites (anterior and posterior arm, anterior and posterior thigh, and posterior lower leg). Bland‐Altman plots (systematic and proportional bias, random error, and 95% limits of agreement), Pearson's product–moment correlation coefficient (*r*), and paired samples t‐tests with Cohen's d effect sizes (ES) were used to assess the concurrent validity of the Lumify device. Systematic bias was low at all sites ( ≤ 0.11 cm) while proportional bias was detected only at the posterior lower leg (*r* ^*2*^ = 0.217 [*r* = 0.466]). The difference in MT between devices was significant only at the anterior thigh (*p* < 0.05); however, ES for all sites were considered trivial (ES ≤ 0.131). Linear associations were found between the devices at each site of measurement (*r* ≥ 0.95). These results highlight that the Lumify probe can be used interchangeably with the Vivid S5 for MT measurements, providing practitioners and researchers with a more cost‐effective and portable alternative for field‐based assessments.

## 1 INTRODUCTION

Muscle size (and therefore mass) is crucial for physical function (Janssen, 2006; Wang et al., 2020), health (Morley et al., 2014), and competitive success in physique sport (Homer et al., 2024), as well as benefitting performance in traditional sports (Hornsby et al., 2018). Change in skeletal muscle size is a critical outcome in assessing adaptation to resistance training (RT) which can be determined by a variety of methods, such as B‐mode ultrasound imaging (Blazevich et al., 2003). Muscle thickness (MT) measurements (Dupont et al., 2001; Juul‐Kristensen et al., 2000), as well as estimations of muscle volume (Miyatani et al., 2002, 2004) and mass (Abe et al., 1997; Sanada et al., 2006) derived from measures of MT have been validated against magnetic resonance imaging (MRI). While MRI is more sensitive than ultrasound in estimating changes in muscle size via MT measurements, similar conclusions between ultrasound MT and MRI‐derived muscle cross‐sectional area measurements highlight the utility of ultrasound imaging in evaluating longitudinal muscle size changes (Loenneke et al., 2019). Nonetheless, with the development of broad variations in the technology, understanding the bounds of their utility for practice is important.

While ultrasound offers a time‐effective, safe, and noninvasive measure of muscle architecture, high‐end devices are expensive and generally constrained to a singular location due to their lack of transportability, limiting their application in the field (Ritsche et al., 2022). In contrast, portable hand‐held devices such as Lumify (Philips Healthcare) offer a more cost‐effective and portable alternative to stationary devices, thus enhancing the accessibility of field testing. Ultimately, these portable devices have the potential to enhance body composition assessments in the field as ultrasound MT measurements are more sensitive to changes in muscle size than commonly implemented surface anthropometry techniques (Gentil et al., 2020).

The Lumify device has been examined in the measurement of gynaecological structures (Toscano et al., 2020) and optic nerve sheath diameter in simulation models (Johnson et al., 2022), and was found to be comparable to a diagnostic ultrasound unit and a laptop‐associated portable unit, respectively. Further, this device has been used in plastic surgery (Miller et al., 2018) and in the education of medical students who were novice scanners (Drake et al., 2021). Most recently, Ritsche et al. (2022) conducted a comparative study between the handheld Lumify and a high‐end laboratory ultrasound device. Within this study, muscle architecture measurements between devices were comparable at some sites and measurements, with MT having the highest agreement (intra‐class correlation coefficient [ICC] = 0.82–0.89) (Ritsche et al., 2022). While this is an important first step in the validation of the Lumify device, assessment of other muscle groups, particularly of the upper body is required to comprehensively evaluate the validity of the device in measuring MT.

To our knowledge, the validity of MT measurements using the Lumify device for both the upper and lower limb has not been investigated. Examination of the upper limb is important as MT values at these sites are generally smaller than that of the lower limb (Thoirs & English, 2009), which may influence the agreement between devices. Furthermore, comparisons at a larger number of sites may identify any potential biases unique to each measurement site. This is relevant for both researchers and practitioners as valid portable probes would allow simplified data collection procedures for evaluating cross‐sectional information on MT and evaluating longitudinal changes in muscle size. Additionally, the replication of previous results is of importance to enhance generalizability across different populations. Therefore, the aim of this investigation was to determine the concurrent validity of the portable Lumify probe and its agreement with the GE Vivid S5 (General Electric Company) for MT measurements of both the upper and lower limbs within a resistance‐trained population.

## 2 METHODS

### 2.1 Ethics approval

Ethical approval for this research was granted by the Auckland University of Technology Ethics Committee (AUTEC reference number: 20/282). Sample size calculations were unable to be conducted as the maximum allowed difference for ultrasound MT values for the measurement sites of interest could not be determined from the literature. Therefore, participants were recruited for this study via purposive convenience sampling based on the specific inclusion and exclusion criteria. The inclusion criteria required participants to be above the age of 18 and currently engaging in regular RT while the exclusion criteria ensured that participants had no existing injury or physical disorder at the sites of measurement. Additionally, participants refrained from vigorous physical activity in the 24 h before data collection. A total of 18 participants (nine males and nine females, Table 1) completed the study following the provision of their written informed consent.

**Table 1:** Participant demographics.

Sex	*N*	Age (years)	Height (cm)	Body mass (kg)
Male	9	26 ± 5.85	180.22 ± 6.01	87.84 ± 9.95
Female	9	24.89 ± 5.79	169.13 ± 7.73	63.87 ± 6.47
Total	18	25.44 ± 5.68	174.67 ± 8.81	75.86 ± 14.59

*Note*: Age, height, and body mass are presented as mean ± standard deviation.

### 2.2 Study design

A repeated‐measures design was used to assess the concurrent validity of the Lumify device and evaluate its agreement with a stationary ultrasound device in measuring MT at five different muscle sites. Testing for each participant was completed in one visit to the laboratory, where anthropometrics was recorded followed by the marking of measurement sites. Subsequent ultrasound imaging of the five measurement sites using both devices, was conducted in the following order: anterior upper arm, anterior thigh, posterior arm, posterior thigh, and posterior lower leg. Three images were captured by each device at each site, from which the means were used to assess the agreement between devices.

### 2.3 Data acquisition and preparation

**Figure 1:**
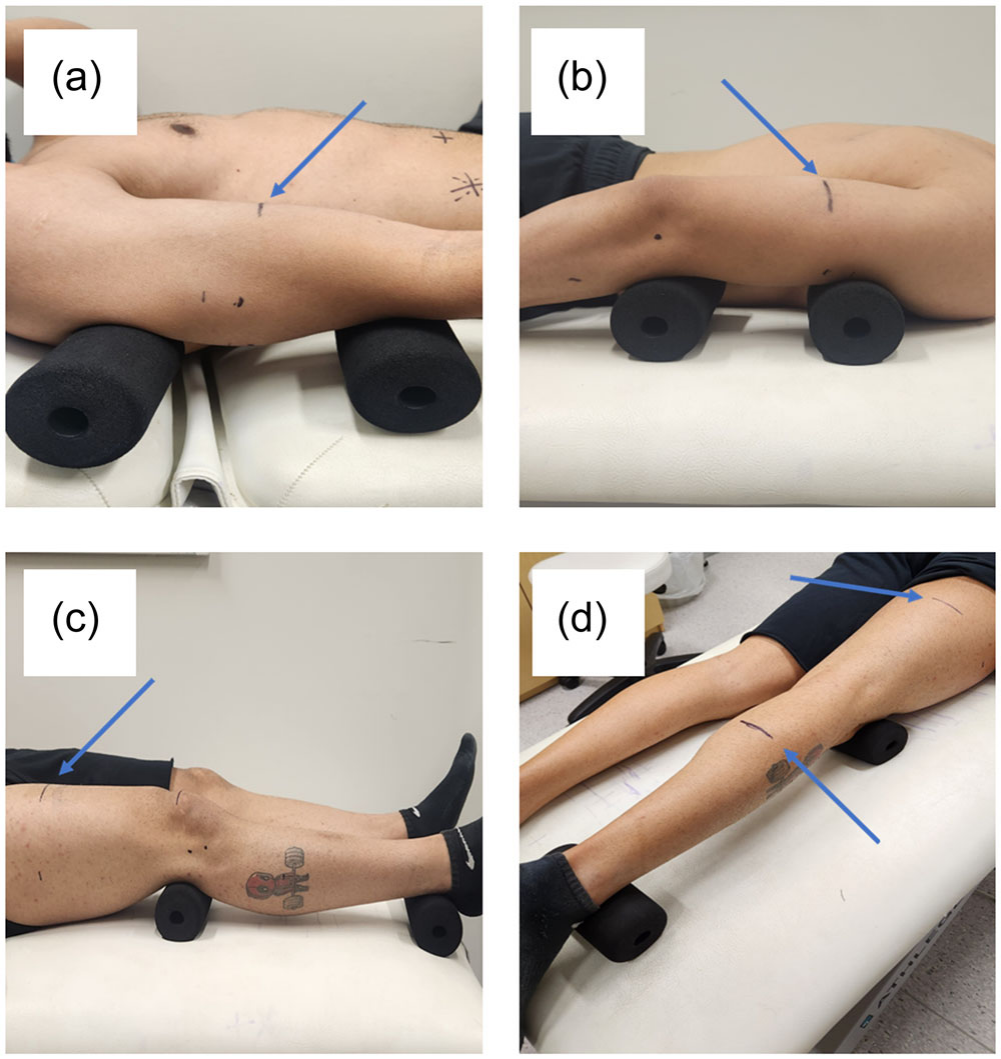
Limb positioning for each of the scanning sites. The scanning sites are as follows: (a) anterior upper arm, (b) posterior upper arm, (c) anterior thigh, and (d) posterior thigh and posterior lower leg. Blue arrows indicate the exact scanning site. Please refer to Table 2 for specific detail on limb positioning.

To evaluate the concurrent validity of the Lumify probe, the stationary Vivid S5 was used as the comparative device as it has been employed to assess MT within recent research (Helms et al., 2023; Oranchuk et al., 2020). Importantly, Oranchuk et al. (2020) found the Vivid S5 to be reliable when assessing MT at different regions of the quadriceps across multiple days (ICC = 0.88–0.98, CV = 2.4%–9.3%). Images on the Lumify were scanned using the L12‐4, linear‐array 37 mm probe set on the Musculoskeletal application. A Samsung Galaxy Tablet S7 FE (Samsung, Seoul, South Korea) was used as the display for the Lumify probe via a wired USB‐C connection through the Lumify application (version 4.04). For the Vivid S5 device, the 12L‐RS, linear 48 mm probe with frequency set to 10 MHz was used. Gain/depth at each site was not standardized between devices and adjusted by the operator as desired. However, these settings were kept consistent within each device for the three images captured at each site. Measures of MT were obtained in the transverse plane and were based on those assessed previously (Abe et al., 1994; English et al., 2012; Sanada et al., 2006; Thoirs & English, 2009), as described and displayed in Table 2 and Figure 1, respectively.

**Table 2:** Measurement sites and limb positioning for ultrasound muscle thickness measurements.

Measurement site		Image plane	Location	Limb positioning
1	Anterior upper arm	Transverse plane over the anterior humeral shaft	Distal to the acromion process of the shoulder, 60% of the measured distance between the acromion process of the scapula and the lateral epicondyle of the humerus	In supine, elbow extended with foam rollers placed beneath the most proximal and distal parts of the humerus (within the armpit and beneath the triceps brachii tendon, respectively)
2	Posterior upper arm	Transverse plane over the posterior humeral shaft		In prone, elbow extended with foam rollers placed beneath the most proximal and distal parts of the humerus (within the arm pit and beneath the cubital fossa, respectively)
3	Anterior thigh	Transverse plane over the anterior femoral shaft	Distal to the greater trochanter, 50% of the measured distance between the most superior edge of the greater trochanter and the most distal prominence of the lateral condyle of the femur	In supine, knee extended with foam rollers placed beneath the popliteal fossa and the Achilles tendon
4	Posterior thigh	Transverse plane over the posterior femoral shaft		In prone, knee extended with foam rollers placed beneath the quadriceps tendon and the talocrural joint
5	Posterior lower leg	Transverse plane over the posterior fibula shaft	Distal to the lateral condyle of the tibia, 30% of the measured distance between the most superior prominence of the lateral condyle of the tibia and the most inferior prominence of the lateral malleolus of the fibula	

*Note*: Measurement site, image plane, and location as adapted from Thoirs and English (2009). Measurements were collected in the following order: anterior upper arm, anterior thigh, posterior upper arm, posterior thigh, and posterior lower leg.

Participants reported to the laboratory following >24 h without vigorous physical activity. The height and body mass of each participant were obtained upon presentation using a wall‐mounted stadiometer (Harpenden Stadiometer; Holtain Limited) and a scale (HW‐200KGL; A&D), respectively. Following this, the exact sites of measurements for each participant were determined by an International Society for the Advancement of Kinanthropometry (ISAK) certified practitioner. The sites were marked using a permanent marker, where a horizontal line with a vertical check line was marked to guide the orientation and positioning of the probe along the line to maximize the accuracy of measurements between devices. Each limb was elevated with two foam rollers (diameter =7 cm, length =15 cm) in accordance with the methodology described in Table 2 to ensure that the musculature of interest was relaxed during the measurements. All images for this study were obtained independently by the same operator.

A water‐soluble transmission gel was applied to the probe which was then placed on the check line marked on the skin. Images were taken in triplicate in the transverse plane with the transducer placed on the skin with the least amount of pressure required to obtain a clear image. Measurements at all five sites were taken with one device first in the same order for each participant, before repeating the measurements with the other device. To counterbalance and account for any order effects, the Lumify probe was used first for the first nine participants and second for the other nine. Anterior measurements were collected first in a supine position before the participant was positioned in a prone position for the posterior measurements.

**Figure 2:**
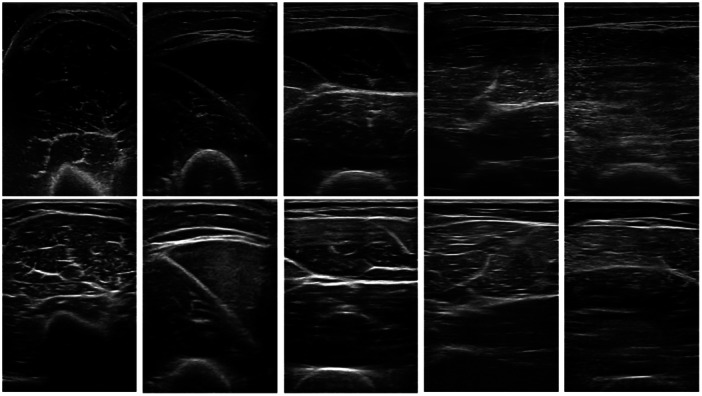
Ultrasound images acquired by the Vivid S5 and Lumify. The top row are images acquired by the Vivid S5, and the bottom row are images acquired by the Lumify. Sites of measurement from Left to Right for both rows: anterior upper arm, posterior upper arm, anterior thigh, posterior thigh, and posterior lower leg.

Captured images saved on each device were exported for independent analysis in ImageJ (version 1.8.0_172) (Schneider et al., 2012) by the same operator. Images were analysed in a randomized order, where a scale was manually set for each image by superimposing a line onto the scale before measurement. MT was determined as the linear vertical distance from the apex (the most superficial pixel) of the periosteum to the deep fascia (the deepest pixel) superficial to the muscle tissue of interest and deep to the subcutaneous layer. The mean of all three measurements for each site was calculated and used for subsequent analysis. Examples of the images acquired by both devices can be seen in Figure 2.

### 2.4 Statistical analysis

Data for MT are presented as means and SD. The normal distribution of the data was confirmed using the Shapiro–Wilk test (*p* > 0.05). Paired samples t‐tests with Cohen's *d*, Bland–Altman plots (systematic bias, random error, and 95% limits of agreement [LoA]) and Pearson's product–moment correlation coefficient (*r*) were used to assess the concurrent validity of the Lumify device. A regression line of the differences was also fitted to each Bland‐Altman plot to detect any proportional bias (Giavarina, 2015), which was determined as being present when *r* ^*2*^ ≥ 0.1 (Atkinson & Nevill, 1998; Pérez‐Castilla et al., 2019). Where any proportional bias was detected with this method, the data at that site was logarithmically transformed before calculating *r* (Atkinson & Nevill, 1998). The criterion to interpret ES was trivial ( < 0.2), small (0.2–0.6), moderate (0.6–1.2), large (1.2–2), and very large (>2) (Pérez‐Castilla et al., 2021), while significance for the paired t‐tests was determined as *p* < 0.05. For LoA, bias, *r*, and ES, 95% CI were calculated to aid interpretation. Statistical analyses were performed in R language and environment for statistical computing (version 4.2.2, The R foundation for Statistical Computing) and with a custom spreadsheet (Hopkins, 2000). Bland–Altman and correlation plots were constructed using the *SimplyAgree* (Caldwell, 2022) and *ggplot2* (Wickham, 2016) packages, respectively. The data set and custom R scripts utilized for this study are available at the Open Science Framework repository (https://osf.io/uft87/).

## 3 RESULTS

**Figure 3:**
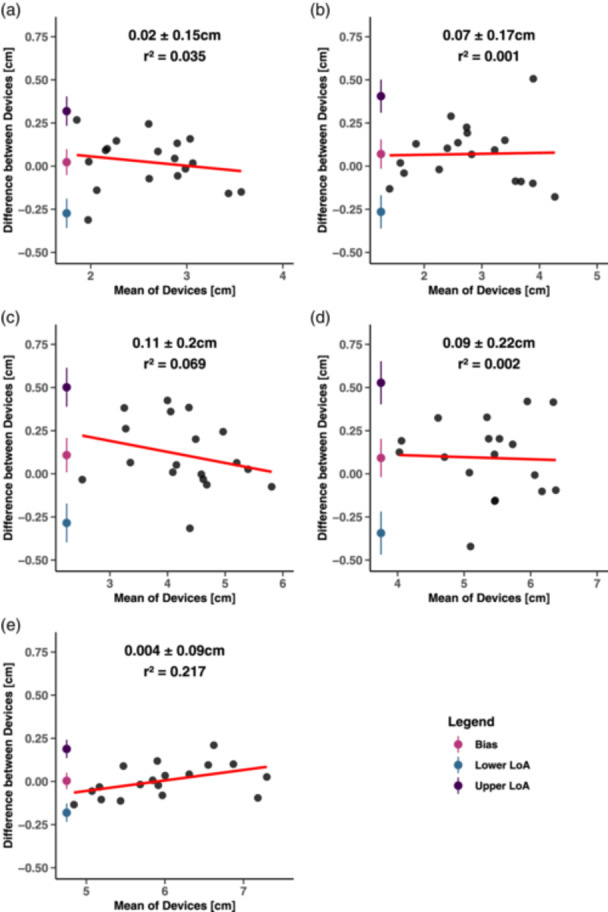
Bland–Altman plots displaying the measurement of muscle thickness between the Vivid S5 and Lumify at each measurement site. Plots of the measurement sites are as follows: (a) anterior arm, (b) posterior arm, (c) anterior thigh, (d) posterior thigh, and (e) posterior lower leg. Systematic bias ± random error, 95% LoA with 95% CI, the regression line, and *r* ^*2*^ are presented within each plot. CI, confidence interval; LoA, limits of agreement.

**Figure 4:**
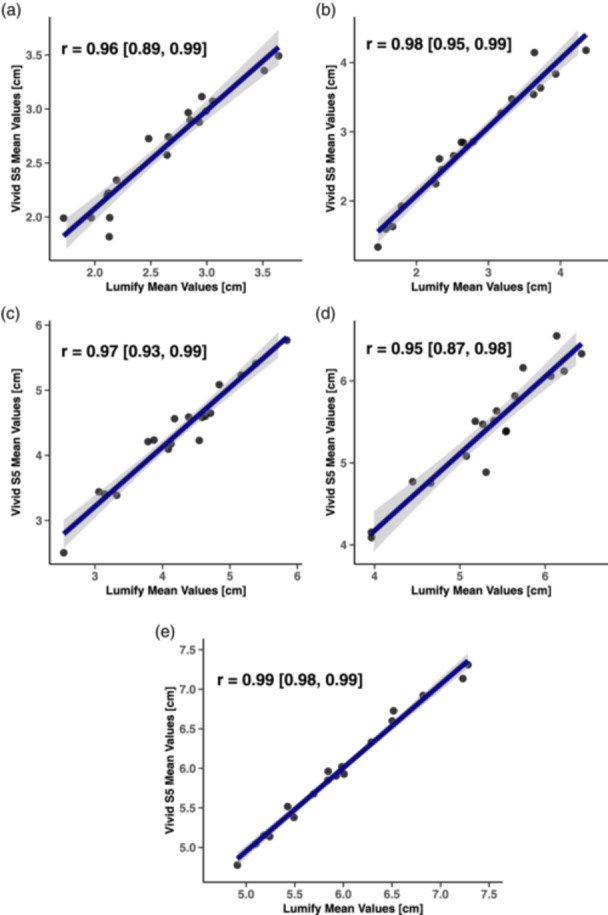
Correlation of muscle thickness measurements between the Vivid S5 and Lumify at each measurement site. Plots of the measurement sites are as follows: (a) anterior arm, (b) posterior arm, (c) anterior thigh, (d) posterior thigh, and (e) posterior lower leg. The linear regression line (blue) and Pearson's product–moment correlation coefficient (*r*) with 95% confidence interval (grey shaded area) are displayed within each plot.

Three replicate measurements were obtained at each site, resulting in a total of 270 observations per device across the 18 participants. Participant age, height, and body mass are provided in Table 1. The mean and SD of MT measurements in addition to statistical parameters (bias, LoA, ES, and *p*‐values) are presented in Table 3. Bland–Altman plots displayed low systematic bias ( ≤ 0.11 cm) and random errors ( ≤ 0.22 cm) across all sites (Figure 3). LoA were the widest for measurements collected at both the anterior and posterior thigh while the LoA were most narrow at the posterior lower leg where proportional bias was also detected (*r* ^*2*^ = 0.217 [*r* = 0.466]). Measurements of MT between the Vivid S5 and the Lumify were linear (*r* ≥ 0.95) at all sites (Figure 4). Paired t‐tests revealed that MT between the devices differed only at the anterior thigh (*p* < 0.05), although the magnitude of difference across all sites was trivial (ES ≤ 0.13).

**Table 3:** Parameters estimated by Bland–Altman and statistical parameters to evaluate the agreement between muscle thickness measurements of the Vivid S5 and Lumify.

Measurement Site	Vivid S5 mean ± SD (cm)	Lumify mean ± SD (cm)	Mean bias (cm) [95% CI]	Lower LoA (cm) [95% CI]	Upper LoA (cm) [95% CI]	ES (*d*) [95% CI]	*p*
Anterior upper arm	2.63 ± 0.51	2.61 ± 0.54	0.02 [−0.05, 0.09]	−0.27 [−0.36, −0.19]	0.32 [0.23, 0.4]	0.04 [−0.09, 0.18]	0.531
Posterior upper arm	2.84 ± 0.87	2.77 ± 0.87	0.07 [−0.02, 0.16]	−0.27 [−0.36, −0.17]	0.41 [0.31, 0.5]	0.08 [−0.01, 0.18]	0.099
Anterior thigh	4.34 ± 0.8	4.23 ± 0.86	0.11 [0.01, 0.21]	−0.29 [−0.4, −0.17]	0.5 [0.39, 0.61]	0.13 [0.01, 0.24]	0.035
Posterior thigh	5.43 ± 0.7	5.33 ± 0.71	0.09 [−0.02, 0.2]	−0.34 [−0.47, −0.22]	0.53 [0.4, 0.65]	0.13 [−0.02, 0.28]	0.097
Posterior lower leg	5.97 ± 0.75	5.96 ± 0.7	0.004 [−0.04, 0.05]	−0.18 [−0.23, −0.13]	0.19 [0.14, 0.24]	0.005 [−0.05, 0.06]	0.869

*Note*: *p*‐Value was determined with a paired samples *t*‐test.

Abbreviations: CI, confidence interval; ES, effect size; LoA, limits of agreement; SD, standard deviation.

## 4 DISCUSSION

This study assessed the concurrent validity of the Lumify L4‐12 portable probe in measuring MT at five different sites. The agreement between Lumify and Vivid S5 devices were lowest at the anterior and posterior thigh (i.e., wider LoA, and significant differences between means for the anterior thigh) while being the highest at the posterior lower leg (i.e., narrowest LoA). Proportional bias, which could signal concern, was detected at the posterior lower leg; however, as the LoA were narrow at this site (lower CI of the lower LoA = −0.23 cm; upper CI of the upper LoA = 0.24 cm), and the absolute differences between devices trivial, this bias may be practically irrelevant. It is important to acknowledge, however, that the differences in measurements between the devices may increase with larger MT values. Positive linear associations were observed, indicating a stable relationship between MT measurements from the devices across all sites. Finally, the differences in MT between the devices were trivial at each muscle site. Therefore, the Lumify portable probe appears to be a valid device that is largely interchangeable with the stationary Vivid S5 in measuring MT.

Ultimately, we observed high levels of agreement between the Lumify and the Vivid S5 at all sites of measurements, in which agreement was the greatest at the posterior lower leg. This may have been a consequence of the site being measured after the posterior thigh for which positioning was shared. Since the posterior lower leg was held in position for longer than the other sites, the increased accuracy of measurements at this site may be partially explained by increased time for intramuscular fluid shifts. Interestingly, proportional bias was also detected at the posterior lower leg, which may be a result of the site having the highest MT values, leading to a greater potential for measurement error (i.e., manual scaling and measurement in ImageJ), especially for larger values of MT. However, the lowest *r* ^*2*^ value was found at the posterior thigh (*r* ^*2*^ = 0.002 [*r* = −0.039]), which contained the second highest mean MT values, indicating that proportional bias may not be present in all large muscle groups. While proportional bias is of concern for practitioners, the low absolute differences between devices for all measurements at the posterior lower leg (ranging from −0.13 to 0.21 cm) and trivial ES (ES = 0.005 [−0.05, 0.06], *p* > 0.05) may render this bias practically irrelevant at this site. Further research is required to determine if proportional bias in the Lumify is present at other sites not examined in the current study.

To the best of our knowledge, this is the second study which has investigated the agreement between the Lumify and a stationary ultrasound device in measuring MT and the first to include the musculature of the upper body. Previously, Ritsche et al. (2022) evaluated the agreement between the Lumify and Acuson Juniper for muscle architecture measurements at three different lower limb sites. For measurements of MT, the bias reported by Ritsche et al. (2022) at their measurement sites (ranging from −0.02 to 0.03 cm) was lower than those found at ours (0.02–0.11 cm), except for the posterior lower leg (0.004 cm). While MT was assessed by both Ritsche et al. (2022) and ourselves, distinct differences in methodology between studies may have led to slightly different results. As Ritsche et al. (2022) assessed muscle architecture (i.e., MT, fascicle length, and pennation angle), images were scanned longitudinally while our images were captured in the transverse plane. Ritsche et al. (2022) also estimated MT using the semi‐automated SMA tool which uses the mean distance between the two superficial aponeuroses of the entire image for measurements (Seynnes & Cronin, 2020). Thus, only the superficial muscles were evaluated, whereas we manually measured the linear distance from the bone to the aponeurosis, including both deep and superficial muscles. Finally, the comparison device employed by Ritsche et al. (2022) was newer than ours which may have contributed to differences in findings. Notwithstanding these differences, 95% LoA was similar between studies except for the posterior lower leg (difference between lower and upper LoA = 0.36 cm) in our study, which was again considerably lower than our other sites of measurement. The difference between lower and upper LoA for our other four sites ranged from 0.6 to 0.87 cm, while those reported by Ritsche et al. (2022) were between 0.49 and 0.79 cm. Thus, indicating similar degrees of agreement between Lumify and the stationary device employed in each study for measuring MT (Myles & Cui, 2007).

While this research provides further evidence of the validity of the Lumify in measuring MT at different lower‐ and upper‐body muscle sites, further research could strengthen the validity and reliability of the device. Namely, the determination and implementation of a practically meaningful threshold corresponding to the smallest detectable change in MT may allow for a more robust and relevant evaluation of the agreement in MT measurements between Lumify and valid stationary ultrasound devices. Further, as only young, healthy adults who engaged in regular RT were recruited for this study, the results may not be wholly generalizable to other populations, particularly those with more subcutaneous and/or less muscle tissue (e.g., clinical and elderly populations). Additionally, the test‐retest reliability of either probe was not assessed within the present study, meaning that the consistency of measurements of the Lumify could not be assessed or compared to Vivid S5. Finally, as the same operator may not always be available, the evaluation of interrater reliability would further the device's utility for practitioners.

## 5 CONCLUSIONS

High levels of agreement between the Lumify and the Vivid S5 in the measurement of MT were found at five different muscle sites. The Lumify measurements were also correlated with the criterion Vivid S5 at all sites, indicating the concurrent validity of the Lumify. Proportional bias was detected at the posterior lower leg; however, given the small absolute differences between devices and trivial ES, this bias may be practically irrelevant. Further research is required to determine if proportional bias within the Lumify is present in any other (large) muscle groups. Overall, it can be concluded that the Lumify probe is interchangeable with the Vivid S5 in making MT measurements at the five sites assessed when averaging across repeated measures. Thus, it appears to be an appropriate instrument for practitioners and researchers collecting MT measurements, providing a more cost‐effective and transportable alternative to laboratory‐based devices, and simplifying field‐based data collection procedures.

## CONFLICT OF INTEREST STATEMENT

The authors declare no conflict of interest.

## Data Availability

The associated data set for this study is available at the Open Science Framework Repository (URL: https://osf.io/uft87/).
